# Educational value of mixed reality combined with a three-dimensional printed model of aortic disease for vascular surgery in the standardized residency training of surgical residents in China: a case control study

**DOI:** 10.1186/s12909-023-04610-9

**Published:** 2023-10-27

**Authors:** Weihao Li, Yuanfeng Liu, Yonghui Wang, Xuemin Zhang, Kun Liu, Yang Jiao, Xiaoming Zhang, Jie Chen, Tao Zhang

**Affiliations:** 1 Department of Vascular Surgery, Peking University People’s Hospital, Beijing, 100044 PR China; 2 Department of Vascular and Endovascular Surgery, First Affiliated Hospital of Zhengzhou University, Zhengzhou, 450052 PR China; 3 Department of Critical Care Medicine, Peking University People’s Hospital, Beijing, 100044 PR China; 4 Beijing Renxin Medical Technology Co., Ltd, Beijing, 100041 PR China; 5 Department of Medical Quality Management, Peking University People’s Hospital, Beijing, 100044 China

**Keywords:** Mixed reality, Three-dimensional printed model, Standardized residency training, Vascular surgery, MiSSES

## Abstract

**Background:**

The simulated three-dimensional (3D) printed anatomical model of the aorta, which has become the norm in medical education, has poor authenticity, tactility, feasibility, and interactivity. Therefore, this study explored the educational value and effect of mixed reality (MR) combined with a 3D printed model of aortic disease in training surgical residents.

**Method:**

Fifty-one resident physicians who rotated in vascular surgery were selected and divided into traditional (27) and experimental (24) teaching groups using the random number table method. After undergoing the experimental and traditional training routines on aortic disease, both the groups took a theoretical test on aortic disease and an assessment of the simulation based on the Michigan Standard Simulation Experience Scale (MiSSES) template. Their scores and assessment results were compared. The study was conducted at the Department of Vascular Surgery of Peking University People’s Hospital, Beijing, China.

**Results:**

In the theoretical test on aortic disease, the experimental teaching group obtained higher mean total scores (79.0 ± 9.1 vs. 72.6 ± 7.5, P = 0.013) and higher scores in anatomy/ pathophysiology (30.8 ± 5.4 vs. 24.8 ± 5.8; *P* < 0.001) than the traditional teaching group. The differences in their scores in the differential diagnosis (25.8 ± 3.0 vs. 23.3 ± 4.9; P = 0.078) and treatment (22.5 ± 11.8 vs. 24.5 ± 8.2; P = 0.603) sessions were insignificant. The MR-assisted teaching stratified the vascular residents through the MiSSES survey. Overall, 95.8% residents (23/24) strongly or somewhat agreed that the MR was adequately realistic and the curriculum helped improve the ability to understanding aortic diseases. Further, 91.7% residents (22/24) strongly or somewhat agreed that the MR-assisted teaching was a good training tool for knowledge on aortic diseases. All residents responded with “Good” or “Outstanding” on the overall rating of the MR experience.

**Conclusions:**

MR combined with the 3D printed model helped residents understand and master aortic disease, particularly regarding anatomy and pathophysiology. Additionally, the realistic 3D printing and MR models improved the self-efficacy of residents in studying aortic diseases, thus greatly stimulating their enthusiasm and initiative to study.

**Supplementary Information:**

The online version contains supplementary material available at 10.1186/s12909-023-04610-9.

## Background

Aortic diseases include conditions such as chronic aortic aneurysms, acute aortic syndrome (AAS), and congenital aortic abnormalities [1]. Most thoracic and abdominal aortic aneurysms are caused by degenerative diseases or atherosclerotic lesions, resulting in the dilatation of the aorta. AAS consists of aortic dissection, intramural hematoma, and penetrating atherosclerotic ulcer with similar clinical characteristics. Common congenital abnormalities include the coarctation of the aorta and many arch variants [2]. Acute events of aortic diseases, such as the rupture of aneurysms, AAS, or massive hemorrhage, are generally fatal, requiring rapid diagnosis and decision-making by vascular surgeons to reduce the extremely poor prognosis [3]. This renders the teaching of aortic diseases one of the most crucial sections of residency training in vascular surgery.

According to the standardized residency training system in mainland China, as established in 2014, surgical residents are required to complete 33 months of training in different departments of surgical medicine in an accredited program regardless of their surgical specialties. Vascular surgery, generally as a part of general surgery, is assigned one month. Vascular surgery education is aimed at equipping residents with mastery of the diagnoses and treatments of common diseases, such as dissection and aneurysm, arteriosclerosis obliterans, acute arterial ischemia, varicosity, and venous thrombus embolism. Of these, teaching about aortic disease is the focus of our department. Anatomical atlases and universal models, computed tomography (CT) angiography images, bedside physical examination of typical patients, and simple schematic diagrams are mainly employed in the traditional teaching approach. Three-dimensional (3D) desktop-based systems can display the shape of the cardiovascular systems of real patients. Thus, physicians and trainees can better elucidate anatomical abnormalities via 3D desktop-based systems, which compensate for the shortcomings of universal models. However, 3D desktop-based systems has its limitations, including limited visualization and absence of interaction opportunities [4].

Mixed reality (MR) refers to new visual environments that combine the real and virtual worlds, where physical and digital objects coexist and interact in real time [5]. Dissimilar to traditional user interfaces, the users of MR are immersed and can interact with 3D models rather than only viewing a screen. A few studies have evaluated the validity of virtual reality, an analogous computer modeling and simulation technology with MR, in medical education. Vuthea Chheang et al. introduced a collaborative virtual reality environment to assist liver surgeons in tumor surgery planning, which allows surgeons to define and adjust virtual resections on patient-specific organ 3D surfaces and 2D image slices, and enables collaborative planning [4]. Katerina Bogomolova et al. developed a virtual 3D assessment scenario for anatomical education. Students and teachers with HoloLens shared the same stereoscopic 3D augmented reality model for anatomical knowledge assessment with real-time interaction between assessor and examinee [6]. However, a systematic review drew no conclusive findings on comparing virtual reality to other available study materials regarding communication skills or clinical decision making [7].

In the era of COVID-19, bedside teaching was greatly hampered. Given the limited bedside teaching opportunities, the vivid explanation of vascular surgical diseases became a particularly difficult problem in clinical teaching. In this context, MR can create an effective virtual teaching scene with the assistance of a headset. However, few experiences about the use of MR technology in vascular surgery teaching have been published. Therefore, we conducted a study to compare the theoretical performance of surgical residents who used MR technology with that of residents exposed to traditional teaching in the context of aortic diseases. The study was conducted in a teaching hospital in China to examine the outcome of MR application.

## Methods

### Participants

The study design was approved by the ethical committee of Peking University People’s Hospital (approval number: 2017PHB155). We obtained written informed consent from the patients involved in the teaching process. The Department of Vascular Surgery, Peking University People’s Hospital undertakes the task of standardized residency training of surgical residents of vascular surgery, as a part of general surgery, for one month. Due to the COVID-19 pandemic, we developed a 3D printed model combined with a MR session for aortic disease training. A total of 24 general surgery residents with limited experience in vascular surgery—which comprised the experimental teaching group—were recruited between July and December 2020 to receive the MR lecture. After their rotation, all the residents took an academic test, as well as an assessment, which was adapted from the Michigan Standard Simulation Experience Scale (MiSSES) template for evaluating simulations [8]. For comparison, a similar cohort comprising 27 general surgery residents rotating in our department between July and December 2019 was recruited as the traditional teaching group. This teaching group underwent traditional training on aortic diseases employing anatomical atlases and CT angiography images. The selected residents for the study were elective and not offered any incentives; moreover, they were not awarded any grades because of their performances during the rotating session. This case-control study was developed using guidance and explanations from the Strengthening the Reporting of Observational Studies in Epidemiology (STROBE) guidelines.

### Teaching methods

Cases involving aortic aneurysm or dissection were selected for the lessons, and the teaching duration was four weeks (8 h/week). The traditional teaching group employed anatomical atlases and CT imaging data, as well as auxiliary simple schematics, to acquire anatomical knowledge and understand the characteristics of the great vessels of the chest and abdomen, the morphological characteristics and classification of the aorta, the principle of endovascular interventional surgery, and the crucial parameters that must be measured (the artery tumor size, tumor neck length and diameter, tumor neck angle, iliac artery branch diameter, and length). Based on traditional anatomical atlases, the experimental teaching group combined CT image data and 3D printed models to specifically learn the anatomical characteristics of large blood vessels, the morphological characteristics, and classification of aortic diseases, as well as actually measure the endovascular treatment design on the 3D printed model and a variety of relevant parameters in the implementation process. Additionally, the residents in this group wore a MR all-in-one machine to watch the surgical process (Supplementary Video 1).

### MR hologram

**Fig. 1:**
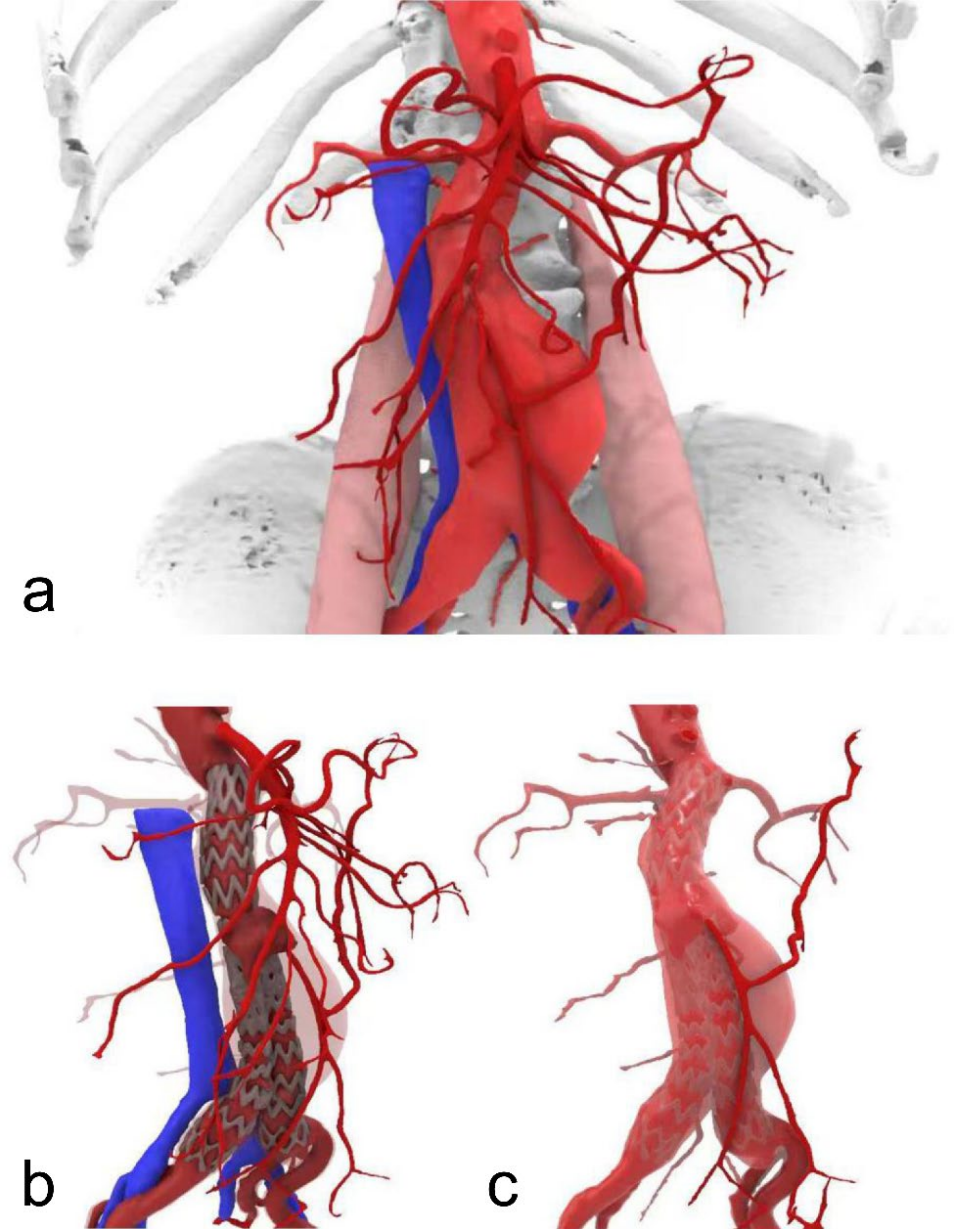
Three-dimensionally reconstructed model of a patient with abdominal aortic aneurysm. The 75-year-old male patient underwent surgery to repair an endovascular aortic aneurysm six months ago, and the review examination revealed an endoleak. This model was employed for learning and evaluation. (A) Abdominal aorta and its primary branches (red), inferior vena cava and the iliac vein (navy blue), bones (white). (B) Endoleak was displayed outside the stent graft. (C) Inferior mesenteric artery revealed patency with the Riolan arch, where blood actively flowed into the aneurysm sac outside the stent graft

All the patients underwent conventional aortic CT angiography, and the scan data were exported in the Digital Imaging and Communications in Medicine (DICOM) format. The first step was to use the 3D reconstruction software (3D Slicer, www.slicer.org) to annotate the CT images of the human body and generate a 3D model of each organ component in STL format. Then, we imported the 3D model in STL format into the 3D rendering software (Rhino, Robert McNeel & Associates), named the organ components, colored the organ components, and set the material characteristics, to export the FBX format files. Thereafter, we imported the 3D model in FBX format into a cross-platform game engine (Unity3D, Unity Technologies), for interactive programming of the 3D model and published the software installation package for Microsoft HoloLens2. Finally, we used software installation package for HoloLens2 to show the holographic model, and interact with the 3D printing models. The facilitating doctor analyzed and taught using the reconstructed images on the holographic image fusion surgery platform based on the patients’ medical records and 3D printing models, emphasizing the areas and structures of interest (Fig. 1, Supplementary Figure S1, and Supplementary Video 2).

### 3D printing

The aortic model in the STL format was acquired, as described above. We employed an open-source 3D printer slicing tool (Cura version_15.04.6, Ultimaker B.V., Netherlands) to transfer the 3D model from an STL format into a printable file (g-code), which was available for the 3D printer. The 3D printed model was created using an Anycubic 4Max Pro printer (Anycubic, Shenzhen, China). The fused deposition modeling (FDM) technique was adopted and polylactic acid (PLA) was utilized as the printing material.

### Test and survey design

The residents were subjected to a specialized theory test on aortic diseases after their rotation, which quantified their understanding of aortic surgery. The test was presented as a case analysis, including several questions on anatomy, pathophysiology, differential diagnosis, and treatment. The full score was 100 points, and the proportions of anatomy and pathophysiology, differential diagnosis, and treatment were ~ 40%, 30%, and 30%, respectively. The test scores were not disclosed to the residents and were not affected by the completeness of the training.

MiSSES is a free online template of a unified assessment instrument that was designed to avail a framework for assessment, offering the option of assessing an entire range of domains that are identified through a review of the simulation literature [8]. We modified the standardized questionnaire of MiSSES for the MR-based teaching method. The adapted scale has been included in the supplemental material (Supplementary file 1). All the residents were required to anonymously complete the adapted MiSSES after their rotations. The collected paper scales were only employed to teach simulation assessment and improve the investigation by the education office of Peking University People’s Hospital.

### Statistical analysis

The data were processed employing PASW Statistics for Windows, Version 18 (SPSS Inc., Chicago, IL, USA). The continuous variables (age, test score, etc.) were presented as mean ± standard deviation and compared with the Student t-test or Mann–Whitney U test, which were determined first by the Shapiro–Wilk test for normality. Furthermore, the MiSSES responses were converted from categorical responses into numerical data for statistical analysis. The residents’ responses, that is, “Strongly disagree,” “Somewhat disagree,” “Neutral/Don’t know,” “Somewhat agree,” and “Strongly agree,” were identified as Scores 1–5 in sequence, respectively. The Cronbach’s alpha value was calculated in aspects to evaluate the reliability of the survey. The categorical variables (gender, answers to closed questions, etc.) were presented as numbers (percentages) and compared with the chi-square test or Fisher exact test, as appropriate. A two-tailed test wherein the level of statistical significance was set at a p-value of < 0.05 was employed for all the statistical analyses.

## Results

A total of 24 residents (14 men and 10 women) aged 23.8 ± 1.0 years completed the 3D printed model combined with the MR all-in-one teaching process. They comprised 10 freshmen in standardized surgical training, 6 residents in the second-year of training, and 8 residents in the final-year of training. All the residents completed MiSSES with a minimum of one answer other than “Don’t know.” A similar demographic characteristic was applied to the traditional teaching group comprising 27 residents who were subjected to traditional teaching (Table 1).

The mean test scores of the experimental and traditional teaching groups were 79.0 ± 9.1 and 72.6 ± 7.5, respectively. The experimental teaching group outperformed the traditional one in the specialized theory test on aortic diseases after their rotations (*P* = 0.013). The experimental teaching group also scored higher in the anatomy and pathophysiology sessions compared to their traditional counterpart (30.8 ± 5.4 vs. 24.8 ± 5.8; *P* < 0.001), while the differences in their scores in the differential diagnosis (25.8 ± 3.0 vs. 23.3 ± 4.9; *P* = 0.078) and treatment (22.5 ± 11.8 vs. 24.5 ± 8.2; *P* = 0.603) sessions were insignificant (Table 1).

**Table 1:** Demographic characteristics and test scores of the two groups of residents

	Experimental teaching group (N = 24)	Traditional teaching group (N = 27)	*P*-value
Gender			0.070
Female	10 (42)	5 (18)	
Male	14 (58)	22 (82)	
Age, years	23.8 ± 1.0	23.4 ± 1.0	0.312
Standardized training			0.492
First-year	10 (42)	9 (33)	
Second-year	6 (25)	11 (41)	
Third-year	8 (33)	7 (26)	
Specialty			0.150
General surgery	15 (63)	9 (33)	
Orthopedics	2 (8)	6 (22)	
Cardio-thoracic surgery	1 (4)	5 (19)	
Urology	4 (17)	6 (22)	
Other	2 (8)	1 (4)	
Test scores	79.0 ± 9.1	72.6 ± 7.5	0.013
Anatomy/pathophysiology	30.8 ± 5.4	24.8 ± 5.8	< 0.001
Differential diagnosis	25.8 ± 3.0	23.3 ± 4.9	0.078
Treatment	22.5 ± 11.8	24.5 ± 8.2	0.603

**Fig. 2:**
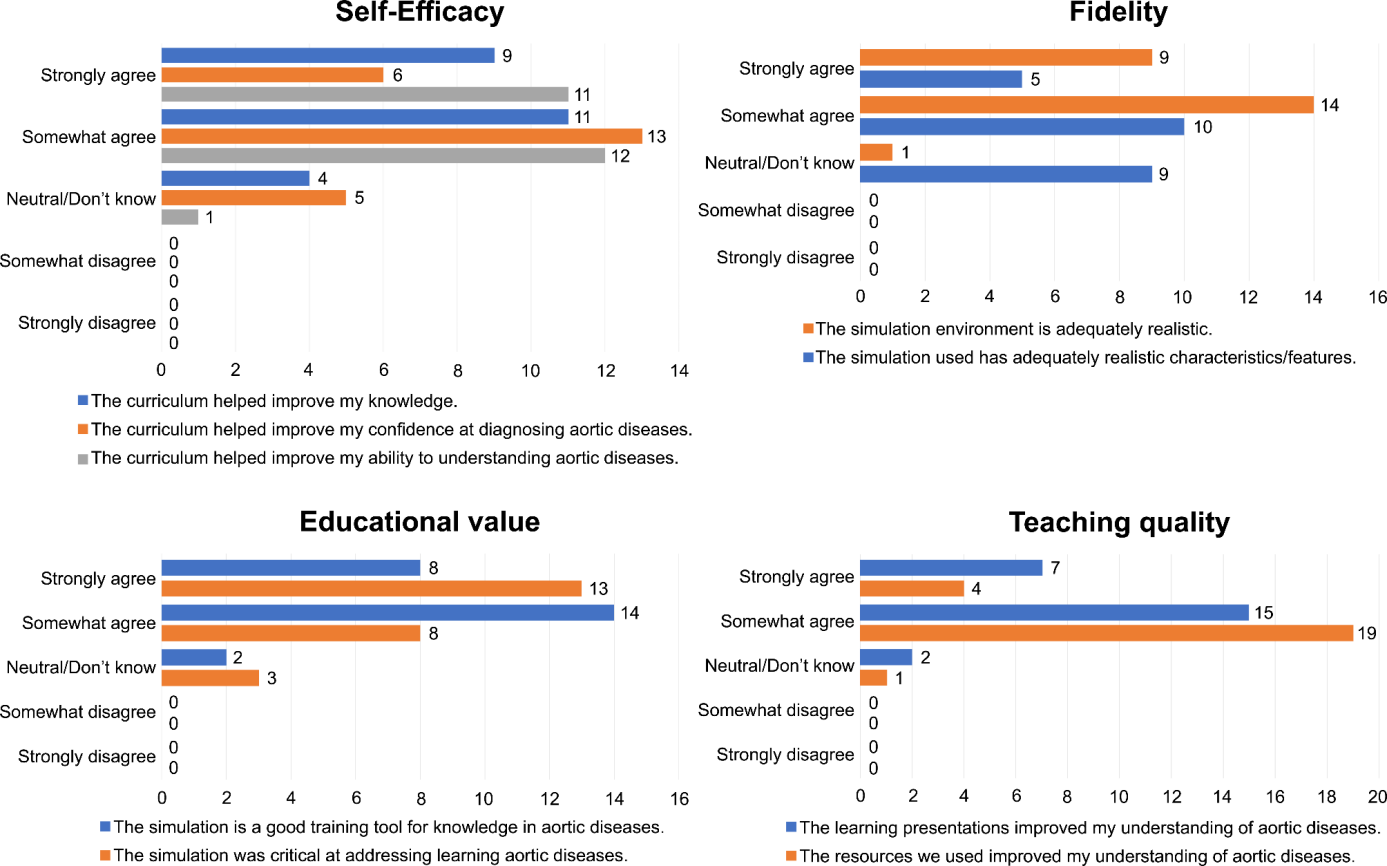
Responses for all the components in the MiSSES survey by the experimental teaching group. All the residents in the experimental teaching group submitted “Neutral” to “Strongly Agree” responses for all the components in the MiSSES survey. More than half of the residents strongly or somewhat agreed that the MR-based lesson with adequately realistic characteristics improves their self-efficacy in learning about aortic diseases. MiSSES, Michigan Standard Simulation Experience Scale; MR, mixed reality

**Fig. 3:**
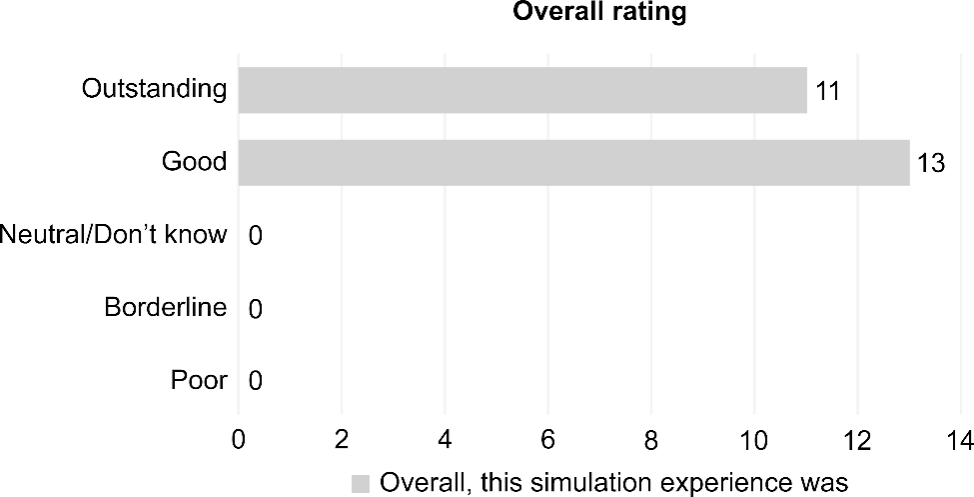
Overall rating from the residents in the experimental teaching group. All the residents responded with “Good” to “Outstanding” on the overall rating of the MR experience, and none rated the experience as “Borderline,” “Poor,” or “Neutral/Don’t Know.” MR, mixed reality

The Cronbach’s alpha value for Self-Efficacy, Fidelity, Educational value, and Teaching quality in MiSSES survey was 0.815, 0.760, 0.702, and 0.698, respectively. The experimental teaching group returned “Neutral” to “Strongly Agree” responses in all the components of the MiSSES survey (Fig. 2, Supplementary Table S1). However, 95.8% residents (23/24) strongly or somewhat agreed that the MR was adequately realistic and it helped improve the ability to understanding aortic diseases. Further, 91.7% residents (22/24) strongly or somewhat agreed that the MR-assisted teaching was a good training tool for knowledge in aortic diseases. All residents satisfied the MR experience with 11 and 13 residents rating it as “Outstanding” and “Good” in the overall rating, respectively (Fig. 3, Supplementary Table S1). No resident rated the experience as “Borderline,” “Poor,” or “Neutral/Don’t Know.”

## Discussion

In this study, surgical residents with limited vascular surgery experience demonstrated significantly high satisfaction in all aspects of the MR and 3D printed model-based lessons, achieving outstanding performances in the concluding test. This finding indicated that the MR-based course positively impacted the vascular surgery residency training of the surgical residents. Beyond the satisfaction reflected by the MiSSES survey, a few (6/24) residents acknowledged that the amazing MR-based course inspired them to pursue a specialty in vascular surgery in the future.

The training of residents with surgical career intentions, certainly including vascular surgery, relies heavily on the interpretation of anatomical structures. However, considering the limited anatomical cadaver resources in China, the surgical residents have only had a very limited exposure to anatomy during their clinical training [9]. Therefore, developing effective modalities for teaching anatomy would be essential for standardized residency training of surgical residents, and the emergence of MR proved as an opportunity for improving students’ knowledge acquisition in clinical medical training by incorporating technology into teaching in the form of computer-generated simulations [10].

The visualized display of the cardiovascular system in the MR-based courses improved the residents’ recognition of anatomical structures, and this improved their understanding of the pathological and pathophysiological mechanisms of aortic diseases. Furthermore, with the transformation of the orientation of current medical education from framed knowledge structure to problem-based teaching, modern medical education concepts emphasize individualized and patient-centered teaching models. Following this concept, MR technology can effectively record and significantly replicate classic cases to rare clinical situations, expose students to different case studies, and enable them to treat many cases more efficiently and rapidly [11].

Interactivity is another notable advantage of the MR technology compared with the 3D printed models or pure CT images. The MR technology offers an anatomically correct and immersive visual–spatial environment, which allows a learner to interact three-dimensionally with the aortic anatomy [12]. The surgical residents controlled the MR system on human body images of the same scales by zooming, focusing, and measuring, as desired, and the teachers also demonstrated aortic diseases in a very intuitive, vivid, and specific manner, which made it easy for the surgical residents to understand and learn the anatomical structure, pathogenesis, diagnosis, and treatment principles of aortic diseases vis a problem-based teaching course. Huettl et al. conducted a study to compare the impact of 3D PDF, 3D printed models, and virtual reality 3D models on anatomical orientation and personal preferences for liver surgeons [13]. Surgeons named significantly more correct segments in virtual reality or 3D printed models compared to PDF. Remarkably, although tumor assignment was significantly shorter with 3D printed models compared to virtual reality application, virtual reality was the most popular method in liver surgeons for its multiple functions such as scaling and transparency adjustment.

In our study, we found a significant improvement: residents in the experimental teaching group had significantly higher test scores than those in the traditional teaching group in the theoretical knowledge test on aortic disease, and their scores in the anatomy and pathophysiology sessions were significantly higher than those of the traditional teaching group. This indicates that MR-based visualization better enabled residents to understand and master aortic disease, particularly regarding anatomy and pathophysiology. Additionally, the realistic MR-based experience improved the self-efficacy of the residents in mastering aortic diseases, thereby significantly stimulating their enthusiasm and initiative for learning. Concurrently, MR can simulate the process of vascular surgery, and this could greatly stimulate students’ enthusiasm and participation in learning. The application space is not limited to the exchange of basic knowledge and skills; it can also be extended to advanced training and exploration of surgical technology.

Our study also has some limitations. First, currently, MR technology cannot provide real tactile feedback, nor can it achieve medical history communication between medical residents and patients, which are irreplaceable advantages of clinical teaching. Secondly, while the presentation of anatomical structures are more pronounced through MR technology, theoretical knowledge requires the dissemination of traditional knowledge point explanation. Thus, we believe that the experimental teaching group performed better in the anatomical/pathophysiological part of the theoretical test, but there was no significant difference in differential diagnosis and treatment principles. In addition, it must be acknowledged that the application of MR technology not only improves students’ ability to understand and accept, but may also impact the teaching ability of clinical teaching doctors. However, in this study, feedback from clinical instructors on the use of MR technology for teaching was not collected. We could examine this in our next work. Further, our study did not have a randomized controlled design; thus, to further confirm the feasibility of MR technology, additional studies with a randomized design in conjunction with more centers need to be conducted.

## Conclusions

We conducted a retrospective controlled study to investigate the value and effect of MR technology combined with the 3D printed model of aortic diseases in residency training education for surgical residents in a teaching hospital in China. Residents who were subjected to MR-based teaching performed well in terms of understanding and mastering aortic diseases, particularly in their understanding of anatomy and pathophysiology. The MR-assisted teaching stratified the vascular residents through the adapted MiSSES survey. We suggest that in the teaching of clinical surgery, especially vascular surgery, more attempts should be made to use techniques such as MR and 3D printing to help students/residents better understand and master complicated surgical diseases.

### Electronic supplementary material

Below is the link to the electronic supplementary material.

Supplementary Material 1

Supplementary Material 2

Supplementary Material 3

Supplementary Material 4

Supplementary Material 5

Supplementary Material 6

Supplementary Material 7

## Data Availability

The datasets used and/or analyzed during the current study are available from the corresponding author on reasonable request.

## Abbreviations

3D: three-dimensional
AAS: acute aortic syndrome
AoD: aortic dissection
DICOM: Digital Imaging and Communications in Medicine
FDM: fused deposition modeling
MiSSES: Michigan Standard Simulation Experience Scale
PLA: polylactic acid
STL: stereolithography
MR: mixed reality
