# Supplementary figures and images for: Educational value of mixed reality combined with a three-dimensional printed model of aortic disease for vascular surgery in the standardized residency training of surgical residents in China: a case control study
Source: BMC Med Educ. 2023 Oct 27;23:812. doi: 10.1186/s12909-023-04610-9 (PMC10612237; doi:10.1186/s12909-023-04610-9)

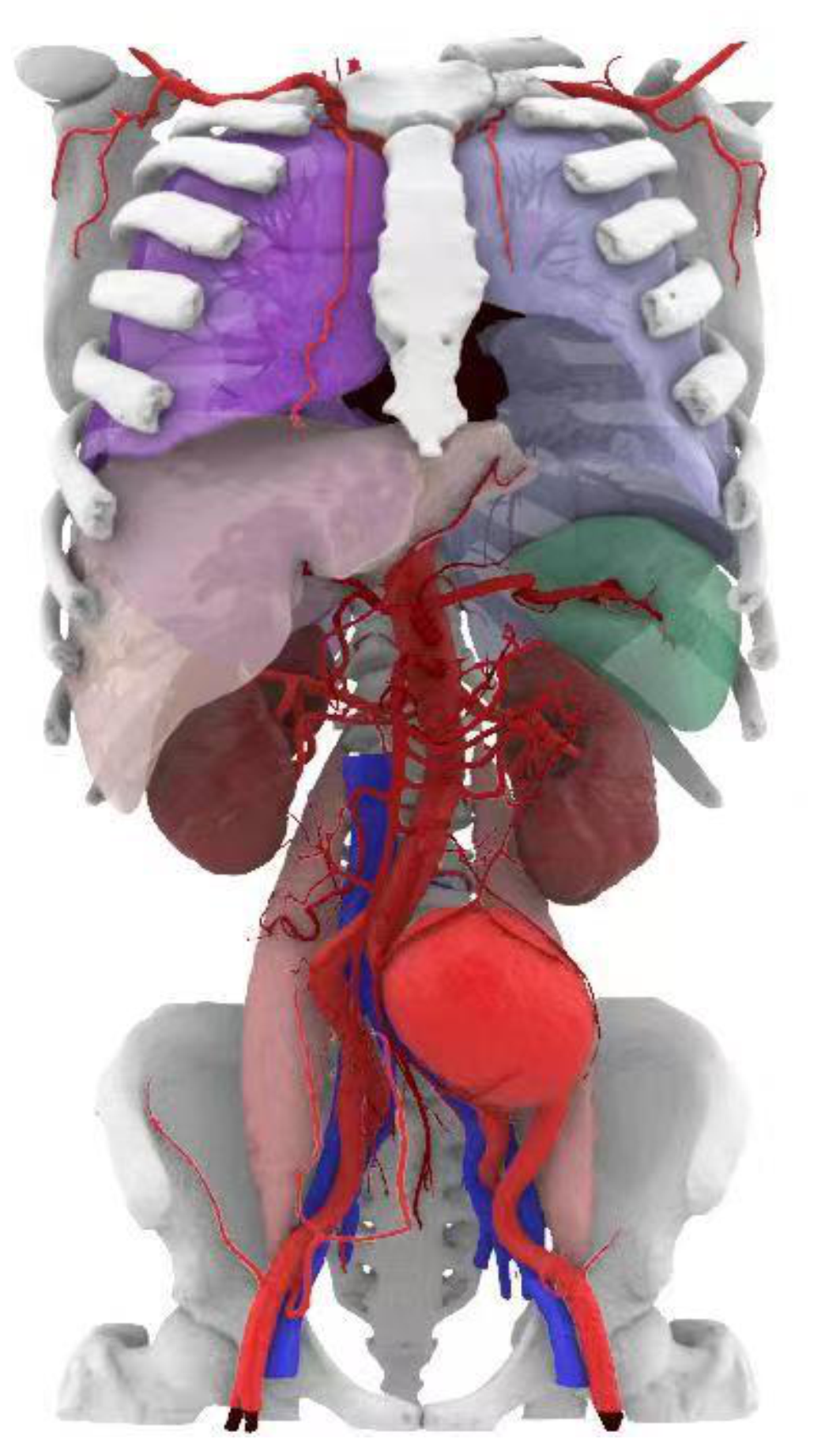

Supplement: Supplementary file 1 — Supplementary Material 1 [file 12909_2023_4610_MOESM1_ESM.tif]

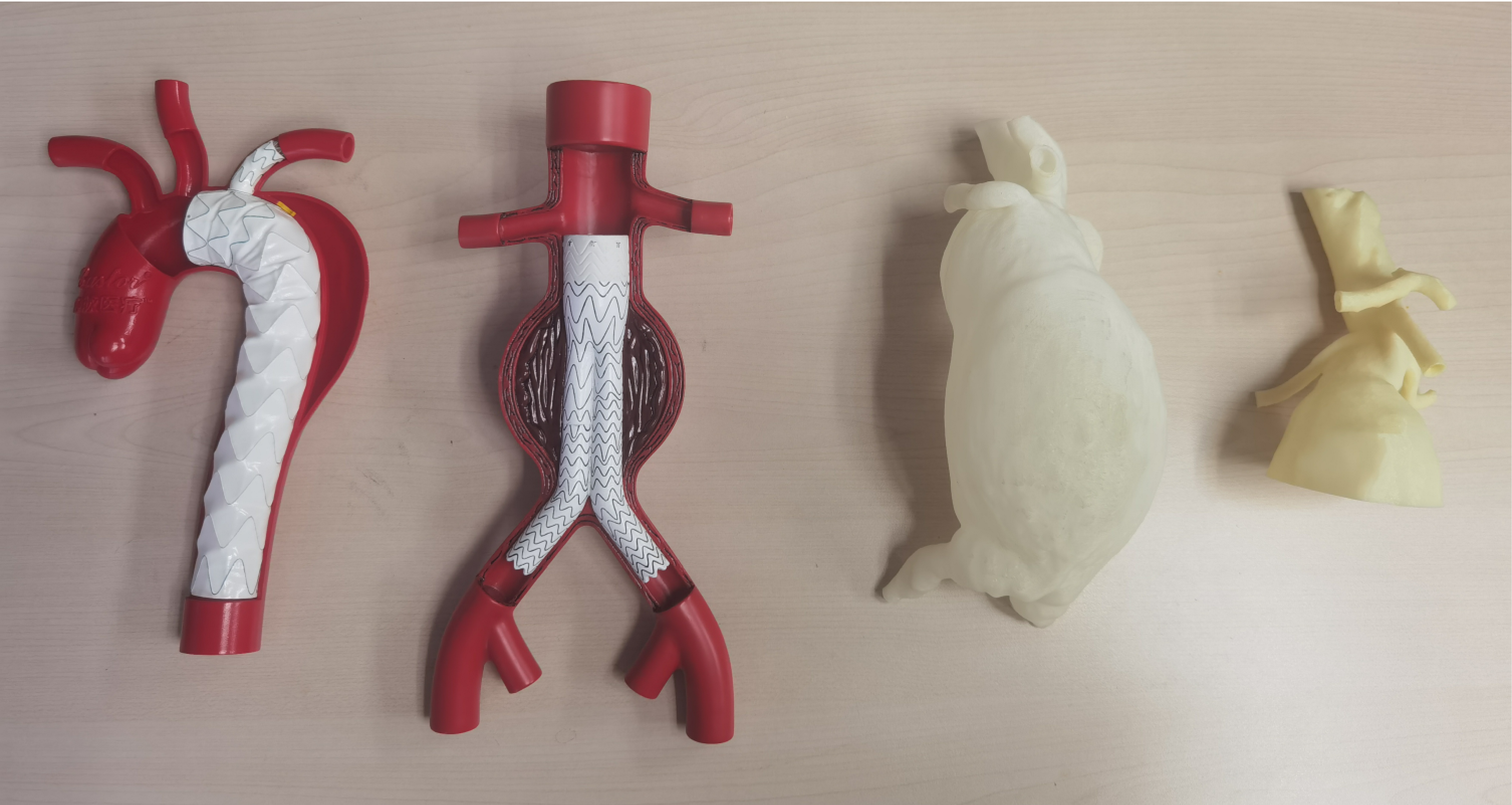

Supplement: Supplementary file 5 — Supplementary Material 5 [file 12909_2023_4610_MOESM5_ESM.tif]
