# Supplementary material for: Educational value of mixed reality combined with a three-dimensional printed model of aortic disease for vascular surgery in the standardized residency training of surgical residents in China: a case control study
Source: BMC Med Educ. 2023 Oct 27;23:812. doi: 10.1186/s12909-023-04610-9 (PMC10612237; doi:10.1186/s12909-023-04610-9)
Supplement: Supplementary file 4 — Supplementary Material 4 [file 12909_2023_4610_MOESM4_ESM.docx]

**Supplementary File Legends**

**Supplementary Table S1.** MiSSES survey results of the experimental teaching group

| Items | Score (N=24) |
| --- | --- |
| SELF-EFFICACY |  |
| The curriculum improved my knowledge. | 4.2±0.7 |
| The curriculum improved my confidence at diagnosing aortic diseases. | 4.0±0.7 |
| The curriculum improved my ability to understanding aortic diseases. | 4.4±0.6 |
| FIDELITY |  |
| The simulation used has adequately realistic features. | 4.3±0.6 |
| The simulation environment is adequately realistic. | 3.8±0.8 |
| EDUCATIONAL VALUE |  |
| The simulation is a good training tool for knowledge in aortic diseases. | 4.3±0.6 |
| The simulation was critical at addressing learning aortic diseases. | 4.4±0.7 |
| TEACHING QUALITY |  |
| The learning presentations improved my understanding of aortic diseases. | 4.2±0.6 |
| The resources we used improved my understanding of aortic diseases | 4.4±0.7 |
| OVERALL RATING |  |
| Overall, this simulation experience was | 4.5±0.5 |

Strongly disagree with 1 point. Somewhat disagree with 2 points. Don’t know/Neutral with 3 points. Somewhat agree with 4 points. Strongly agree with 5 points.

**Supplementary Figure S1**. Three-dimensionally reconstructed model of a patient with iliac aneurysm: arteries (red), veins (navy blue), bones (white), lungs (purple), liver (gray), spleen (green), and kidney (deep red)

**Supplementary Figure S2**. The traditional teaching molds of aortic aneurysm (the left 2 molds), and the 3D printing model of aortic aneurysm (the right 2 molds)

**Supplementary Video 1**. The use of virtual reality model to teach the residents

**Supplementary Video 2**. Interactable virtual reality display model. The teachers and residents could control the elements to deepen their understanding of anatomy

**Supplementary file 1.** The adapted questionnaire of MiSSES for the MR-based teaching method

**Supplementary file 2**. The specialized theory test on aortic diseases.
