# Supplementary material for: Educational value of mixed reality combined with a three-dimensional printed model of aortic disease for vascular surgery in the standardized residency training of surgical residents in China: a case control study
Source: BMC Med Educ. 2023 Oct 27;23:812. doi: 10.1186/s12909-023-04610-9 (PMC10612237; doi:10.1186/s12909-023-04610-9)
Supplement: Supplementary file 6 — Supplementary Material 6 [file 12909_2023_4610_MOESM6_ESM.doc]

**DEMOGRAPHICS**

**Training Year (please circle):** ZZY Y 1 2 3

**Specialty** (please fill in blank or Circle N/A): ________________________________ N/A

**Age:** _____

**Gender** (please circle)**:** Male Female Other

**Previous experience:** Approximately how many aortic surgery procedures have you performed? _____________

Please take a moment to complete the survey below. Your feedback will guide improvements for the aortic disease curriculum.

|  | Don’t know  (.) | Strongly disagree  (1) | Somewhat disagree  (2) | Neutral  (3) | Somewhat agree  (4) | Strongly agree  (5) |
| --- | --- | --- | --- | --- | --- | --- |
| **Self-Efficacy** | | | | | | |
| The curriculum improved my **knowledge.** |  |  |  |  |  |  |
| The curriculum improved my **confidence** at diagnosing aortic diseases. |  |  |  |  |  |  |
| The curriculum improved my **ability** to understanding aortic diseases. |  |  |  |  |  |  |
| Comments/suggestions regarding the curriculum that may improve your self-efficacy: | | | | | | |
| **Fidelity** | | | | | | |
| The simulation used has adequately realistic features. |  |  |  |  |  |  |
| The simulation environment is adequately realistic. |  |  |  |  |  |  |
| Comments/suggestions regarding the simulation to improve the fidelity during the training session. | | | | | | |
| **PLEASE CONTINUE ON THE NEXT PAGE 🡪** | | | | | | |
|  | Don’t know  (.) | Strongly disagree  (1) | Somewhat disagree  (2) | Neutral  (3) | Somewhat agree  (4) | Strongly agree  (5) |
| **Educational Value** | | | | | | |
| The simulation is a good training tool to gain **knowledge** regarding aortic diseases. |  |  |  |  |  |  |
| The simulation was critical in addressing the learning of aortic diseases. |  |  |  |  |  |  |
| Comments regarding educational value | | | | | | |
| **Teaching QUALITY** | | | | | | |
| The learning presentations improved my understanding of aortic diseases. |  |  |  |  |  |  |
| The resources we used improved my understanding of aortic diseases |  |  |  |  |  |  |
| Comments regarding teaching quality: | | | | | | |
| **Overall RAting** | | | | | | |
| Overall, this simulation experience was |  |  |  |  |  |  |

| **Global- Please check *one* statement below with which you most agree.**  **For evaluation of simulator:**  □ This simulation requires **extensive improvements** before it can be considered for use in training.  □ This simulation requires **minor adjustments** before it can be considered for use in training.  □ This simulation can be used in training, but **should be improved slightly**.  □ This simulation **can be used in training** with no improvements.  Please suggest any changes you would make to the simulator.  What specific changes would you suggest for improving your learning experience? Please use the space provided below to describe the same. |
| --- |
