# Supplementary material for: Educational value of mixed reality combined with a three-dimensional printed model of aortic disease for vascular surgery in the standardized residency training of surgical residents in China: a case control study
Source: BMC Med Educ. 2023 Oct 27;23:812. doi: 10.1186/s12909-023-04610-9 (PMC10612237; doi:10.1186/s12909-023-04610-9)
Supplement: Supplementary file 7 — Supplementary Material 7 [file 12909_2023_4610_MOESM7_ESM.docx]

**Case analysis**

Mr. Unknown, male, 38 years old, was brought to the emergency department of the hospital mainly due to "sudden back pain and abdominal pain for six hours." The patient had no obvious trigger for sudden severe back pain six hours ago from the top to the his lower back, followed by abdominal cramps that continued to worsen without relief. However, the patient did not experience nausea or vomiting. He felt soreness in the left lumbar, accompanied by coldness, pain, and movement disorders in both lower limbs. He was taken to the emergency room by ambulance. The patient displayed a failure to urinate and defecate after onset. He was addicted to cigarettes and alcohol. There was no history of sudden death among his immediate family members.

His body temperature in the emergency room was 37.5 °C, breathing was 32 breaths per minute, pulse was 60 beats per minute, and blood pressure was 186/112 mmHg. He was overweight and had a pained face, but did not have jaundice. He had abdominal hardness, positive periumbilical tenderness, and rebound tenderness. Bowel sounds were not heard. The bilateral radial pulse was symmetrical, but the bilateral femoral pulse was absent. The bilateral foot was pale, cool, and paralyzed.

**Questions:**

1. Please give a primary diagnosis of this patient and the basis for the diagnosis. (10 points)

2. What are the possible causes of coldness, pain, movement disorders in both lower limbs? Why? (15 points)

3. What tests are required to confirm the diagnosis? Please briefly describe the significance of these tests. (15 points)

4. What are the causes of acute back pain? Please list at least three causes. (10 points)

5. What are the causes of acute abdominal pain? Please list at least five causes. (20 points)

6. What is the principle line of treatment for this patient? (30 points)
